# Trophic structure of a nektobenthic community exploited by a multispecific bottom trawling fishery in Northeastern Brazil

**DOI:** 10.1371/journal.pone.0246491

**Published:** 2021-02-08

**Authors:** Alex Souza Lira, Flávia Lucena-Frédou, Frédéric Ménard, Thierry Frédou, Júlio Guazzelli Gonzalez, Valdimere Ferreira, José Souto Rosa Filho, Jean-Marie Munaron, François Le Loc’h

**Affiliations:** 1 Departamento de Pesca e Aquicultura, Universidade Federal Rural de Pernambuco (UFRPE), Recife, Pernambuco, Brazil; 2 IRD, Univ Brest, CNRS, Ifremer, LEMAR, F-29280 Plouzané, France; 3 Aix Marseille Univ, Univ Toulon, CNRS, IRD, MIO, UM110, Marseille, France; 4 MARBEC, Univ. Montpellier, CNRS, IRD, Ifremer, 34095, Montpellier, France; 5 Departamento de Oceanografia, Laboratório de Bentos (LABEN), Universidade Federal de Pernambuco, Recife, Pernambuco, Brazil; Universita del Salento, ITALY

## Abstract

We used complementary stable isotope (SIA) and stomach content (SCA) analyses to investigate feeding relationships among species of the nektobenthic communities and the potential ecological effects of the bottom trawling of a coastal ecosystem in northeastern Brazil. Carbon (δ^13^C) and nitrogen (δ^15^N) compositions were determined for five basal sources and 28 consumers, from zooplankton to shrimp and fish species. Fishes and basal sources showed a broad range of δ^15^N (fishes: 6.49–14.94‰; sources: 2.58–6.79‰) and δ^13^C values (fishes: -23.86 to -13.71‰; sources: -24.32 to -13.53‰), while shrimps and crabs exhibited similar nitrogen and carbon ratios. Six trophic consumer groups were determined among zooplankton, crustaceans and fishes by SIA, with trophic pathways associated mostly with benthic sources. SCA results indicated a preference for benthic invertebrates, mainly worms, crabs and shrimps, as prey for the fish fauna, highlighting their importance in the food web. In overall, differences between SCA and the SIA approaches were observed, except for groups composed mainly for shrimps and some species of high δ^15^N values, mostly piscivorous and zoobenthivores. Given the absence of regulation for bottom trawling activities in the area, the cumulative effects of trawling on population parameters, species composition, potentially decreasing the abundance of benthic preys (e.g., shrimps, worms and crabs) may lead to changes in the trophic structure potentially affect the food web and the sustainability of the fishery.

## Introduction

Bottom trawling impacts marine habitats in three main aspects: i) physical, due to direct changes in the seabed structure [1], causing the resuspension of sediment (sediment’s matrix disruption) and injury or death of many benthic organisms [2–4]; ii) chemical, affecting the organic carbon mineralization [5,6] and re-inserting into the water column possible contaminants such as mercury [7]; and iii) biological, mainly given its high level of non-targeted catch [8–10], mostly composed of small sized individuals, usually juveniles [11,12].

In the food web, the fishing activity may act as regulator of the ecosystem, causing adverse ecological effects that could lead to major changes in the trophic interactions among species, consequently to marine habitat degradation [13–16]. Particularly concerning the bottom trawling, direct food web effects are associated to the reduction of species richness and abundance [17–19], however, important indirect consequences are usually disregarded [20]. The capture of non-targeted species by bottom trawling may be a potential risk for the ecosystem sustainability, not only by removing predators of high trophic level, but also prey of lower trophic levels, as the untargeted invertebrates [14,21–23]. For example, a decline in prey availability for demersal fishes, could potentially reduce food intake and body condition [24], causing a trophic cascade effect, changing the ecosystem control equilibrium, either top-down or bottom-up, or even reaching the extreme collapse of the ecosystem [25–27]. In this context, the effect of the predator-prey interactions into the ecosystem trophic structure may be accessed, either by the diet composition and natural markers (such as isotope analysis) [28], and also though ecosystem models (such as Ecopath) [29].

One of the traditional and most accessible ways to address the feeding habits of fish species is by qualitative and quantitative Stomach Content Analysis (SCA) [28–30]. However, often when considering spatial and temporal variations, this approach may be misleading, providing only “snapshots” of the diet [31,32]. On the other hand, Stable Isotope Analysis (SIA) is one of the newest ecological tools in diet studies, providing information that are incorporated in the consumer tissues over a longer period of time [33], indicating resources poorly quantified by stomach contents methods due to regurgitation and digestion rates of preys [34,35]. Although less subject to temporal bias, the SIA approach are influenced, for example, by the type of tissue sampled, lipid concentration, climate season, life stage and size spectrum [36–38].

However, even if SIA and SCA are inherently different techniques, both with considerable assumptions and caveats [39], the use of the these approaches as complementary tools, has been largely recommended [40–43]. For example, increases of δ^13^C and δ^15^N may be related to the decrease in the biomass of benthic consumers, while the decrease of biomass of benthic preys causes the reduction in the trophic level of the species [45]. Currently, the assessment of the trawling impacts in the food-wed are restricted to SIA, when evaluating changes in carbon (δ^13^C) and nitrogen (δ^15^N) compositions and the trophic level of consumers or prey, and to SCA when considering the biomass of the preys [44–46].

Although the Brazilian northeastern coast covers an extensive area and encompasses a wide range of environments, few studies of coastal trophic structure have been carried out, often focusing only on describing qualitatively and quantitatively the diet [47–50], and in the functioning of the ecosystem [51–53]. Even of great importance, the probable effect of the “disturbance” in the trophic web by fishing, especially those with high impact in the ecosystem (e.g., bottom trawling), has never been focused. Specifically, in Pernambuco, Northeast Brazil, despite the socio-economic relevance of the shrimp fishery, the activity is completely unregulated. Sirinhaém has the largest and most productive motorized fishing fleet among the coastal cities of Pernambuco, corresponding to 50% of the shrimp catch [54], being extremely important as income source for local population [55].

In this study, we investigated the trophic structure of the nektobenthic community exploited by the shrimp trawl fisheries in the State of Pernambuco, Northeastern Brazil, using stable isotopes (SIA) of carbon and nitrogen and stomach content (SCA) analyses. Our main aim is to determine the importance of the target species (shrimps) as prey for non-target species (bycatch fishes), also discussing the possible effects of the bottom trawling into the trophic interactions, which may affect the marine local community.

## Material and methods

### Study area and field sampling

In the west coast of the South Atlantic Ocean, mainly in Brazil, shrimps are exploited by a multispecies fishery along the entire coastline, mainly in shallow areas with motorized bottom trawl nets [56], being the Penaeidae the main target [57]. Three fishery systems, which differ in size, technology and volume of catch occur in the Brazilian waters: (i) the industrial fleet operating mainly in the North region (Amazon river estuarine system), Southeast and South Brazil; (ii) a semi-industrial fleet distributed from north to south of the country with similar technology of the artisanal fleet but with greater fishing power and catches; and (iii) artisanal fleet that operates along the entire coast, but specially in Northeast, characterized by higher number of people involved; low level of technology, capture and profit [58]. This later fishery system is present in our study area, Sirinhaém. This fishery has the proportion of fish bycatch: shrimp as 0.39:1 kg [59]. The fish bycatch is composed of 51 species, 38 genera and 17 families, primarily Pristigasteridae, Sciaenidae and Haemulidae, mostly zooplanktivore and zoobenthivore (e.g., *Pellona harroweri*, *C*. *bleekerianus*, *Isophistus parvipinnis*, *Stellifer microps*, *Larimus breviceps*, *P*. *brasiliensis*, *C*. *nobilis* and *Haemulopsis corvinaeformis*), which are often used as a byproduct (commercially valuable species) or consumed by the crew and local communities [59].

**Fig 1:**
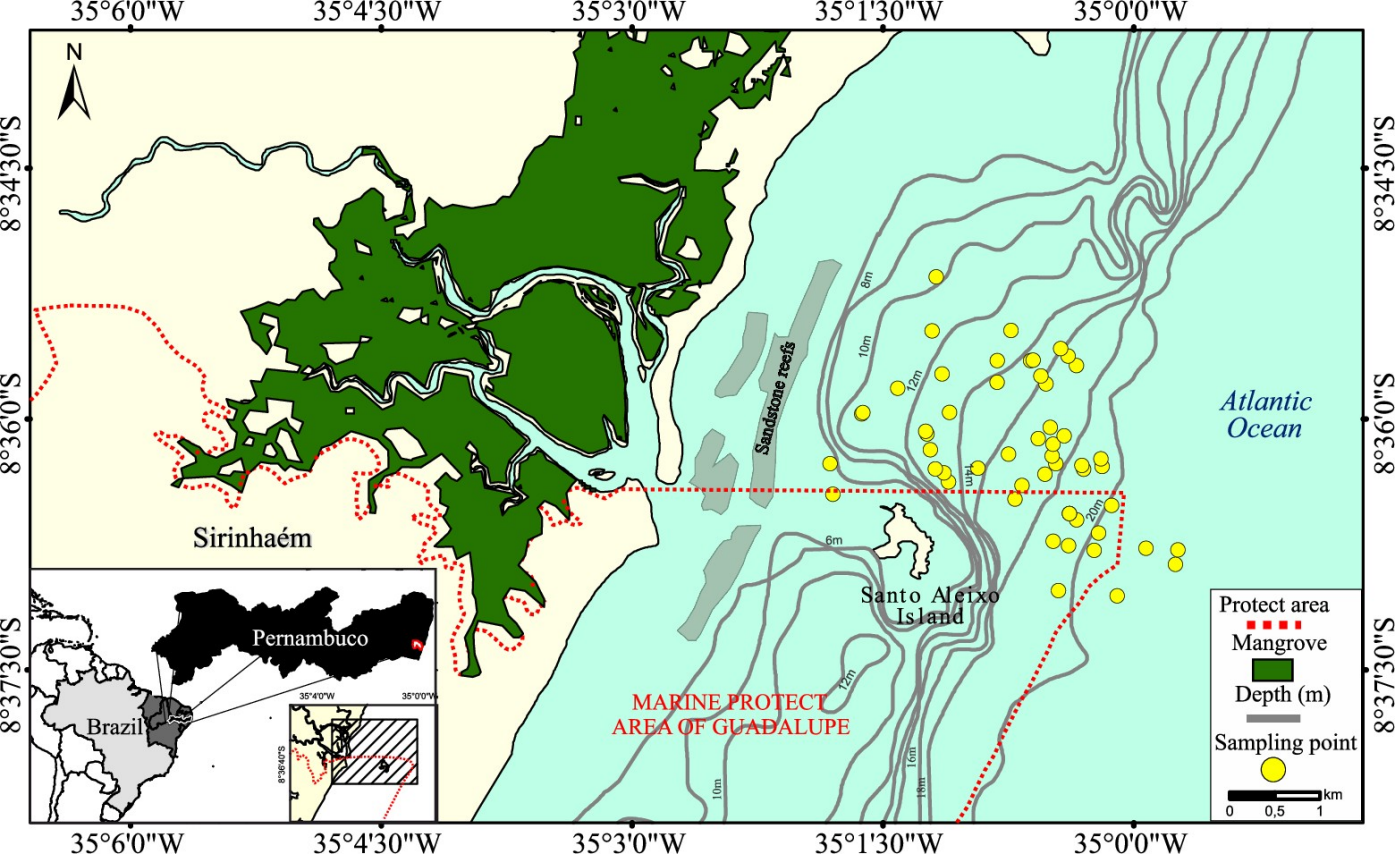
Study area located on the Pernambuco coast in northeast Brazil. The Sirinhaém area, located on the Pernambuco coast in northeast Brazil. Depth was obtained from [63].

The coastal waters are influenced by nutrient supply from the Sirinhaém river, the climate is tropical, with a rainy season occurring between May and October. In terms of environmental condition, the rainfall ranges monthly from 20 to 450 mm·yr^−1^, the mean water surface temperature is 29°C, and the pH and salinity range between 8.0 and 8.7 and 23–37, respectively [60,61]. The shrimp fishery is artisanal and carried out near the coast [62] between 8 and 20 m depth, mainly inside or close to the Marine Protected Area of Guadalupe, around of Santo Aleixo Island, distant from 1.5 to 3 miles off the coast (Fig 1).

Surveys to collect macroalgae, bycatch fishes and invertebrates (except zooplankton) were carried out quarterly with the approval by the Brazilian authorities, such as the Navy and the Ministry of the Environment (Sisbio—License n°34125), between 2014 and 2015 using the commercial bottom trawl fishing (length: 10 m; horizontal opening: 6.10 m; mesh size body: 30 mm; mesh size cod end: 25 mm). It was not required the approval by the Brazilian animal ethics committee, since species collected arrive dead onboard without any method of sacrifice and within the authorized fishery activity. In order to improve the data samples with other consumers of the bycatch not previously sampled, complementary data collections were carried out in October to December 2019 (see S1 Table for detail).

At each month, three trawls were performed during the daytime, between 10 and 20 m depth, for about 2 hours, with boat velocity varying between 1.6 and 3.7 knots. Zooplankton was sampled with a 300 μm mesh size plankton net hauled horizontally for 10 minutes at subsurface. In addition, basal food sources included suspended Particulate Organic Matter (POM) obtained by filtering 0.5–1.0 L of water through fiberglass filters (0.75 μm) and Sediment particulate Organic Matter (SOM) collected at low tide in a shallow area near the island from the top 2 mm layer of sediment using a tube core (2 cm of diameter) [37]. All compartments sampled and specimens caught were at once put on ice, then transported to the laboratory and stored in a freezer (-18°C) until the analysis. In laboratory, they were identified to species level and measured (standard length–SL for fishes and carapace length/diameter for shrimps and blue crabs).

### Data analysis

Muscle samples (about 0.5g) from each fish, squid, blue crab and shrimp species were extracted, rinsed with distilled water to remove exogenous materials (e.g., remaining scales, bones and carapace). For POM, SOM and zooplankton (which comprehended only copepods), the whole organism/sample was used. Samples were dried in an oven at 60°C for 48h. Then, they were ground into a fine powder with a mortar and pestle.

POM, SOM and zooplankton samples were duplicated. The inorganic carbon was removed by acidification process prior to the δ^13^C analysis [64]. The sub-samples that were not acidified were analyzed for δ^15^N [31]. Samples were analyzed by continuous flow on a Thermo Scientific Flash EA 2000 elemental analyzer coupled to a Delta V Plus mass spectrometer at the Pôle Spectrométrie Océan (Plouzané, France). Results are expressed in standard δ notation based on international standards (Vienna Pee Dee Belemnite for δ^13^C and atmospheric nitrogen for δ^15^N) following the equation:

$$\delta^{13}C\;or\;\delta^{15}N=\left[(\frac{R_{sample}}{R_{standard}})-1\right]\times 10^{3}\;(\mathrm{in}‰),\;\mathrm{where}\;R\;\mathrm{is}\;{}^{13}C/{}^{12}C\;\mathrm{or}\;{}^{15}N/{}^{14}N$$ (Eq 1)

Reference materials of known δ^15^N and δ^13^C were analyzed: USGS61, USGS62 and USGS63. The recommended values of the standards were reproduced within the confidence limits. For every six samples, a home standard (Thermo Acetanilide) of experimental precision (based on the standard deviation of the internal standard replicates) was used, indicating an analytical precision of ± 0.11‰ for δ^13^C and ± 0.07‰ for δ^15^N.

The carbon and nitrogen values of basal food sources and consumers of different trophic guilds [65] in Sirinhaém coast were investigated by the biplot of mean δ^13^C (±Standard deviation (SD)) and δ^15^N (±SD) values of each group/species. Due to the non-normality (Kolmogorov-Smirnov test) and non-homogeneity of variance (Bartlett test), the statistical significance of differences between individual δ^13^C and δ^15^N values of food sources, shrimp and fish bycatch species was assessed with the non-parametric Kruskal-Wallis test and pairwise multiple comparisons tested for subsequent comparisons in case of significant differences (*p-value*<0.05) [66].

From the mean values of δ^13^C and δ^15^N (objects) for each consumer species (descriptors), an Agglomerative Hierarchical Cluster (AHC) using the Ward’s minimum variance method based in Euclidian similarity resemblance matrix was performed in order to identify trophic groups of species [67,68]. To determine optimal number of clusters, the NbClust method proposed by Charrad et al. [69] was carried out. This method provides 30 indices to evaluate the relevant number of Clusters. In addition, the trophic groups obtained with AHC were compared using a Nonparametric multivariate permutational analysis of variance (PERMANOVA) [70]. All statistical analyses were performed considering a 5% significance level.

Stomach Content Analysis (SCA) were accessed for 52% of species (13 species, 52% of the total) caught in the same area, including fishes and shrimps from unpublished laboratory database, except *Conodon nobilis* [71]. For the remaining species (12), diet information was obtained from literature and detailed in the Tables 2 and S2. For local collected species, the stomachs were removed and weighed to the nearest 0.01 g and fixed in 10% formaldehyde within 48 h and then conserved in 70% alcohol. The contents of the individual stomachs were sorted, counted, weighed (g), and identified to the lowest possible taxonomic level.

To describe the diet composition of the consumers, the stomach content items were gathered in 9 prey groups (detritus, phytoplankton, zooplankton, worm, crab, mollusk, other crustaceans, shrimp and fish). The similarity of diet among species was accessed by AHC as explained earlier, using prey weight proportion (objects; %W) [55] for each consumer (descriptors).

To provide an overview comparison among SIA and SCA, the stomach contents data was graphically displayed through heatmaps (consumer x prey) along with a AHC, using prey weight proportion (%W) [72] for each consumer. In the heatmap approach, the individual values contained in a matrix were represented as color ramp within a range of %W value scale. In addition, the hierarchical cluster obtained from SIA was compared graphically to SCA and quantified by Baker’s Gamma Index (BGI) with permutation test [73,74] to identify the possible level of similarity among the dendrograms, and consequently the two approaches. BGI value ranges from -1 to 1, values close to 0 represents statistic difference between the two dendrograms (*p*<0.05), and values close to -1 and 1 reveals identical dendrogram.

All analyses were performed using the R environment [75], with packages vegan [76], cluster [77], NbClust [69] and dendextend [73] for the estimation the clusters, to identify the optimum cluster number and to measure the association between the two trees of hierarchical clustering respectively. Additionally, ggplot2 [78] and gplots [79] were used to generate graphics.

## Results

Stable isotope compositions were analyzed in six invertebrate species and eighteen consumers—fish (167 samples), one zooplankton group (6 samples) and five basal sources (31 samples) (Table 1). Fishes and basal sources showed a broad range of δ^15^N (fishes: 6.49–14.94‰; sources: 2.58–6.79‰) and δ^13^C values (fishes: −23.86 to −13.71‰; sources: −24.32 to −13.53‰), while shrimps and *Callinectes* species exhibited similar values of nitrogen and carbon ratios (Table 1).

**Table 1:** Stable isotopes compositions of basal sources and consumers.

Groups/species	Code	Guilds	N	δ^13^C (‰)	Min-Max	δ^15^N (‰)	Min-Max
**Basal sources**							
Sedimentary organic matter	SOM	-	8	-16.51 ± 0.60	[-17.35 to -15.84]	3.67 ± 0.55	[2.85 to 4.37]
*Lobophora variegata*	lob.var	-	6	-15.02 ± 0.84	[-15.74 to -13.53]	4.36 ± 0.44	[3.88 to 4.89]
*Gracilaria cervicornis*	gra.cer	-	6	-21.98 ± 1.92	[-24.32 to -18.63]	4.44 ± 1.09	[3.59 to 6.58]
*Sargassum* sp.	sar.sp	-	6	-17.50 ± 1.41	[-19.34 to -15.69]	4.44 ± 0.24	[4.07 to 4.73]
Particulate organic matter	POM	-	5	-21.60 ± 0.65	[-22.35 to -20.61]	6.39 ± 0.36	[5.90 to 6.79]
**Invertebrates**							
Zooplankton	zoo	Filter-feeder	6	-18.65 ± 0.51	[-19.32 to -17.84]	7.26 ± 1.14	[6.45 to 9.49]
*Penaeus subtilis*	pen.sub	Omnivore	14	-16.71 ± 1.89	[-21.59 to -14.69]	8.83 ± 2.19	[7.38 to 11.72]
*Penaeus schmitti*	pen.sch	Detritivore	20	-16.29 ± 1.18	[-18.45 to -13.60]	8.98 ± 1.51	[6.85 to 11.18]
Callinectes danae	cal.dan	Omnivore	5	-15.14 ± 0.61	[-16.01 to -14.45]	9.07 ± 0.62	[8.52 to 9.75]
Callinectes ornatus	cal.orn	Omnivore	3	-14.87 ± 0.67	[-15.41 to -14.12]	9.27 ± 0.86	[8.47 to 10.18]
*Xiphopenaeus kroyeri*	xip.kro	Omnivore	17	-15.95 ± 0.59	[-17.01 to -15.14]	9.27 ± 0.48	[8.05 to 9.76]
Lolliguncula brevis	lol.bre	Piscivore/Zoobenthivore	5	-16.77 ± 0.17	[-16.91 to -16.58]	12.60 ± 0.10	[12.53 to 12.75]
**Fishes**							
*Citharichthys spilopterus*	cit.spi	Zoobenthivore	3	-21.59 ± 2.65	[-23.86 to -18.68]	8.85 ± 1.59	[7.91 to 10.68]
*Diapterus auratus*	dia.aur	Zoobenthivore	7	-17.52 ± 2.88	[-21.44 to -13.71]	8.84 ± 1.23	[7.74 to 11.47]
*Opisthonema oglinum*	opi.ogl	Zooplanktivore	8	-17.07 ± 0.47	[-17.60 to -16.19]	9.58 ± 1.01	[8.35 to 11.83]
*Symphurus tessellatus*	sym.tes	Zoobenthivore	6	-21.56 ± 1.54	[-23.20 to -19.08]	9.69 ± 1.22	[8.71 to 11.86]
*Diapterus rhombeus*	dia.rho	Zoobenthivore	8	-19.22 ± 2.19	[-22.50 to -17.06]	9.71 ± 1.49	[7.11 to 11.41]
*Lutjanus synagris*	lut.syn	Zoobenthivore	6	-15.74 ± 0.81	[-16.77 to -14.75]	10.21 ± 1.50	[8.71 to 11.76]
*Bairdiella ronchus*	bai.ron	Zoobenthivore	3	-16.02 ± 0.08	[-16.11 to -15.95]	10.54 ±0.1	[10.36 to 10.70]
*Chirocentrodon bleekerianus*	chi.ble	Zoobenthivore	4	-16.84 ± 0.23	[-17.15 to -16.64]	10.59 ± 0.80	[8.28 to 11.81]
*Eucinostomus argenteus*	euc.arg	Omnivore	14	-16.11 ± 1.21	[-18.87 to -14.99]	10.98 ± 1.51	[6.49 to 13.19]
Bagre bagre	bag.bag	Zoobenthivore	3	-16.28 ± 0.09	[-16.38 to -16.21]	11.62 ± 0.40	[11.13 to 11.93]
*Caranx hippos*	car.hip	Piscivore	8	-17.13 ± 1.56	[-19.73 to -15.83]	11.75 ± 0.50	[10.36 to 10.70]
Micropogonias furnieri	mic.fur	Omnivore	7	-16.59 ± 1.32	[-18.18 to -15.12]	12.07 ± 0.60	[11.15 to 12.82]
*Bagre marinus*	bag.mar	Zoobenthivore	8	-16.18 ± 0.23	[-16.59 to -15.84]	12.18 ± 0.70	[11.33 to 13.47]
*Larimus breviceps*	lar.bre	Zoobenthivore	3	-16.29 ± 0.51	[-16.61 to -15.7]	12.19 ± 1.00	[11.18 to 13.18]
*Stellifer microps*	ste.mic	Zoobenthivore	4	-16.26 ± 0.81	[-17.32 to -15.44]	12.21 ± 1.60	[10.40 to 13.64]
*Isopisthus parvipinnis*	iso.par	Piscivore	4	-15.93 ± 0.28	[-16.15 to -15.56]	12.50 ± 0.19	[12.33 to 12.74]
*Conodon nobilis*	con.nob	Piscivore/Zoobenthivore	4	-15.58 ± 0.31	[-15.93 to -15.21]	12.71 ± 1.50	[11.45 to 14.94]
*Paralonchurus brasiliensis*	par.bra	Zoobenthivore	3	-15.20 ± 1.20	[-16.58 to -14.44]	12.89 ± 1.60	[11.23 to 14.45]

Groups/species names, codes, trophic guilds, numbers of samples (n), δ^13^C means ± standard deviation, minimum and maximum, δ^15^N mean ± standard deviation, and minimal and maximum of basal sources and consumers (invertebrates and fishes) sampled off the Sirinhaém coast, northeastern Brazil.

**Fig 2:**
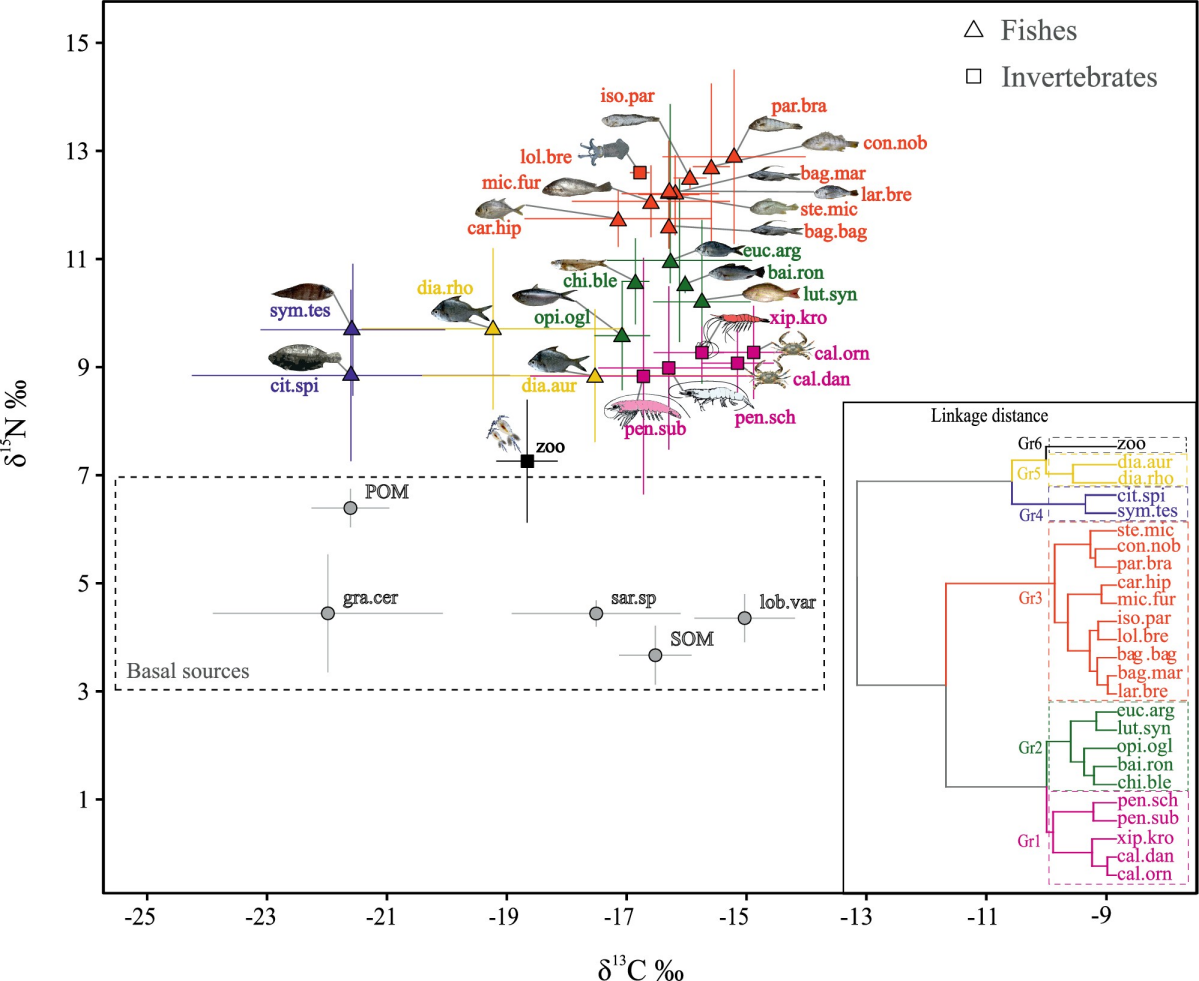
Biplot of carbon and nitrogen for basal sources and consumers. Biplot of δ^13^C (‰) and δ^15^N (‰) values (mean ± SD) for basal sources (grey circles) and consumers (invertebrates and fishes) sampled off the Sirinhaém coast, northeastern Brazil. The dendrogram inserted in the right corner is from agglomerative hierarchical clustering (AHC) for 25 consumers representing the trophic groups, indicated by colours, where each node represents an individual species. Species abbreviations are: Sedimentary organic matter (SOM), *Lobophora variegata* (lob.var), *Gracilaria cervicorni* (gra.cer), *Sargassum* sp.(sar.sp), Particulate organic matter (POM), Zooplankton–(zoo), *Penaeus subtilis* (pen.sub), *Penaeus schmitti* (pen.sch), *Callinectes danae* (cal.dan), *Callinectes ornatus* (cal.orn), *Xiphopenaeus kroyeri* (xip.kro), *Lolliguncula brevis* (lol.bre), *Citharichthys spilopterus* (cit.spi), *Diapterus auratus* (dia.aur), *Opisthonema oglinum* (opi.ogl), *Symphurus tessellatus* (sym.tes), *Diapterus rhombeus* (dia.rho), *Lutjanus* synagris (lut.syn), *Bairdiella ronchus* (bai.ron), *Chirocentrodon bleekerianus* (chi.ble), *Eucinostomus argenteus* (euc.arg), *Bagre bagre* (bag.bag), *Caranx hippos* (car.hip), *Micropogonias furnieri* (mic.fur), *Bagre marinus* (bag.mar), *Larimus breviceps* (lar.bre), *Stellifer microps* (ste.mic), *Isopisthus parvipinnis* (iso.par), *Conodon nobilis* (con.nob) and *Paralonchurus brasiliensis* (par.bra).

Basal sources exhibited significant difference within the medians for both δ^13^C values (Kruskal-Wallis: χ^2^ = 17.814, *p-value* = 0.001) and δ^15^N (Kruskal-Wallis: χ^2^ = 23.668, *p-value* < 0.001) (Fig 2), for example between POM and SOM in δ^15^N, and the macroalgae *Lobophora variegate* and *Gracilaria cervicornis* in δ^13^C. The medians of δ^13^C values for the three shrimp species (*Penaeus subtilis*, *P*. *schmitti* and *Xiphopenaeus kroyeri*) were similar (Kruskal-Wallis: χ^2^ = 1.555, *p-value* = 0.459), as well as for δ^15^N values (Kruskal-Wallis: χ^2^ = 2.6428, *p-value* = 0.266). Significant differences were observed in δ^15^N and δ^13^C values (Kruskal-Wallis: χ^2^ = 63.44, *p-value* < 0.001; χ^2^ = 52.083, *p-value* < 0.001 respectively) for fish species, mostly due to *Citharichthys spilopterus*, *Symphurus tesellatus*, *Eucinostomus argenteus* and *Diapterus auratus* which showed the more depleted δ^15^N and δ^13^C values.

Among the basal sources, POM and SOM had maximum and minimum δ^15^N values respectively (6.79 and 2.85‰), while *G*. *cervicornis* and *L*. *variegata* showed the most depleted and enriched δ^13^C values, respectively (Fig 2). Between consumers, flatfish species (*C*. *spilopterus* and *S*. *tesellatus*) had the most depleted δ^13^C values and blue crab species (*Callinectes danae* and *C*. *ornatus*) were the most enriched. For the δ^15^N rates, zooplankton had the lowest, while *Conodon nobilis*, *Paralonchurus brasiliensis* and *Lolliguncula brevis* showed the highest values (Fig 2).

Cluster analysis performed on mean stable isotope ratio values for the consumer group significantly gathered species in 3 main groups (GR), divided on 2 to 3 sub-groups (Fig 2 inset) (PERMANOVA: F = 49.12; *p-value* < 0.001). Zooplankton, the only member of GR6, had the lowest δ^15^N.

Fish species associated to the seabed had relatively lower δ^13^C compared to the others and were separated into two groups, mojarras (*D*. *rhombeus* and *D*. *auratus*; GR5) and flatfish species (*S*. *tesselatus* and *C*. *spilopterus*; GR4) (Fig 2). The cluster GR3 regrouped the species of highest δ^15^N values, greater than 11‰, as piscivorous and zoobenthivore, while GR2 represented zooplanktivore, omnivore and zoobenthivore fishes of intermediate values of carbon (δ^13^C: -17.04 to -15.74‰) and nitrogen (δ^15^N: 9.58 to 10.98‰) (Fig 2 and Table 1). GR1 gathered the omnivorous or detritivores invertebrates, as shrimp and blue crab, with low δ^15^N values and enriched δ^13^C (Fig 2).

The diet description of the 25 consumers species/groups through SCA may be accessed in Table 2. Omnivorous and detritivores species, including shrimp (e.g., *P*. *schmitti*) and blue crabs (e.g., *C*. *ornatus*), showed high trophic plasticity, feeding from phytoplankton to fishes in proportions ranging, in average, from 8 to 25% for each group of prey (Table 2). Omnivorous fishes (e.g., *E*. *argenteus* and *Micropogonias furnieri*) were an exception, feeding predominantly on benthic fauna, as shrimp and worms, totalizing 60% and 95% of their diet, respectively (Table 2), while *Opisthonema oglinum*, classified as zooplanktivore, fed mainly on phytoplankton and zooplankton, which represented 83% of the diet (Table 2).

**Table 2:** Weight contribution (%) of each prey group in the diet of consumers off the Sirinhaém coast, northeastern Brazil.

Consumers	Weight contribution of preys (%W)									
	Det	Phy	Zoo	Cra	Shr	Wor	Mol	Oth.crus	Fis	Sources
Zooplankton (zoo)	0.15	0.80	0.05							[80]
*Penaeus subtilis* (pen.sub)	0.12	0.08	0.30			0.30		0.20		unpublished data
*Penaeus schmitti* (pen.sch)		0.50	0.06			0.24		0.20		unpublished data
*Callinectes danae* (cal.dan)	0.04			0.26	0.01	0.35	0.23	0.03	0.08	[81]
*Callinectes ornatus* (cal.orn)	0.1	0.04	0.02	0.25	0.18	0.04	0.12	0.02	0.22	[82]
*Xiphopenaeus kroyeri* (xip.kro)	0.22	0.07	0.37	0.03	0.11	0.08	0.05	0.04	0.05	unpublished data
*Lolliguncula brevis* (lol.bre)	0.15	0.01	0.01	0.24	0.24	0.01	0.02		0.32	[83]
Citharichthys spilopterus (cit.spi)			0.09	0.02	0.21	0.29		0.01	0.38	[84]
*Diapterus auratus* (dia.aur)			0.01			0.96	0.01		0.02	unpublished data
*Opisthonema oglinum* (opi.ogl)	0.05	0.42	0.41					0.11	0.01	[85,86]
*Symphurus tessellatus* (sym.tes)			0.31	0.01	0.03	0.66				[84]
*Diapterus rhombeus* (dia.rho)		0.02	0.82			0.16				unpublished data
*Lutjanus synagris* (lut.syn)		0.01	0.16	0.39	0.18	0.05		0.11	0.10	[87]
*Bairdiella ronchus* (bai.ron)	0.04			0.18	0.22			0.26	0.29	unpublished data
*Chirocentrodon bleekerianus* (chi.ble)	0.01		0.12	0.10	0.32	0.01		0.30	0.14	[88]
*Eucinostomus argenteus* (euc.arg)	0.02		0.13	0.03	0.14	0.52		0.02	0.14	unpublished data
*Bagre bagre* (bag.bag)		0.01	0.01	0.21	0.23	0.15			0.39	[89]
*Caranx hippos* (car.hip)	0.02		0.02	0.01	0.15	0.01	0.01	0.22	0.57	unpublished data
*Micropogonias furnieri* (mic.fur)			0.02		0.35	0.60			0.03	[90]
*Bagre marinus* (bag.mar)	0.12	0.03		0.54	0.14	0.01		0.02	0.15	unpublished data
*Larimus breviceps* (lar.bre)	0.03		0.01		0.80	0.16				[91]
*Stellifer microps* (ste.mic)				0.19	0.60	0.02	0.01	0.06	0.02	unpublished data
*Isopisthus parvipinnis* (iso.par)				0.01	0.16			0.01	0.82	unpublished data
*Conodon nobilis* (con.nob)					0.62	0.01			0.31	unpublished data
*Paralonchurus brasiliensis* (par.bra)					0.40				0.01	unpublished data

The values represent the percentage of weight contribution of each prey group. Acronyms for each prey are: Det–Detritus; Phy–Phytoplankton; Zoo–Zooplankton; Cra–Crab; Shr–Shrimp; Wor–Worm; Mol–Mollusc; Oth.cru—Other crustaceans and Fis–Fish.

Shrimps, fishes, and worms were the main preys, contributing on average 50% of the stomach content of fishes and squids (*L*. *brevis*) (Table 2). In this group, *P*. *brasiliensis* was an exception, with a diet composed basically of detritus (58%) and shrimp (40%), similar to detritivorous species. Species classified as piscivores, *Caranx hippos* and *Isopisthus parvipinnis*, presented high percentage of fish in their diet, 82% and 57% respectively (Table 2).

**Fig 3:**
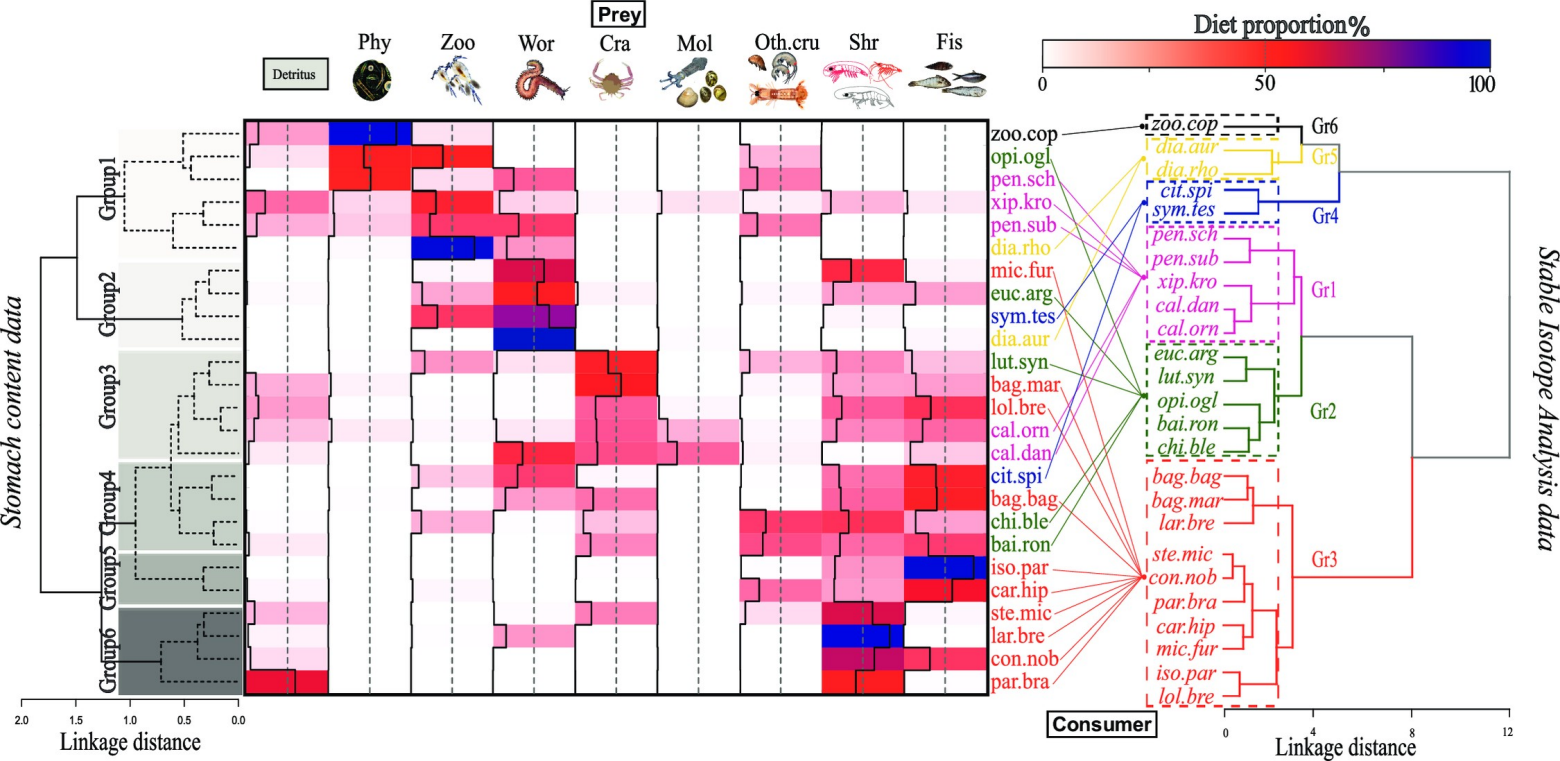
Heatmap of the diet proportion among consumers and prey. The dendrograms inserted in the corners were made with agglomerative hierarchical clustering (AHC) based on diet proportion by stomach content data (left) and isotope composition data (right) off the Sirinhaém coast, northeastern Brazil. The grey boxes represent different groups based on stomach content data. Consumer abbreviations are given in Table 1 and colours based on clustering by isotope composition data. Acronyms for each prey are: Det–Detritus; Phy–Phytoplankton; Zoo–Zooplankton; Cra–Crab; Shr–Shrimp; Wor–Worm; Mol–Mollusc; Oth.cru—Other crustaceans and Fis–Fish.

Cluster analysis of SCA emphasized 6 significantly different main consumer groups (Fig 3) (PERMANOVA: F = 6.50; *p-value* < 0.001). Group 1 (six species) had diet based mainly on detritus, phytoplankton and zooplankton and worms, while the second group was composed of four species (e.g., flatfish and croaker) that fed mainly on worms (Fig 3 left). The group 3 (five species) and group 4 (four species) (e.g., *Bagre marinus*, *Chirocentrodon bleekerianus* and *L*. *synagris*), showed considerable variability in dietary items in the stomach contents dominated by crustaceans and fishes (Fig 3). In the last clusters of two (Group 5) and four species (Group 6), composed by piscivores or zoobenthivore species of high δ^15^N values (Fig 2 and Table 1), the main preys were fish or shrimps (Fig 3).

The species with high δ^15^N values (e.g., *P*. *brasiliensis*, *C*. *nobilis* and *C*. *hippos*), as well as shrimps (*P*. *schmitti*, *P*. *subtilis*, *X*. *kroyeri*) showed a similar grouping between the two approaches (SIA and SCA). However, in overall, differences in diagram clusters between stomach contents and the SIA approach were observed (Baker’s Gamma correlation coefficient = 0.20). Some species presented large grouping differences between the two approaches, mainly for species of the GR4 (e.g., *C*. *spilopterus*, and *S*. *tesselatus*) and zoobenthivores of the GR2 (e.g., *O*. *oglinum*, and *E*. *argenteus*), based in SIA clusters (Fig 3).

## Discussion

The trophic ecology has long been assessed from diet composition to evaluate level of complexity, health and alterations of communities on aquatic ecosystems (e.g., rivers, estuaries, reefs and deep oceans) [47,92–95]. Additional tools as the trophic natural markers provide information on the assimilated food, while the traditional approach of diet composition is based only on food intake. Comparing the two approaches improves the description and potentially minimizes errors in measuring the organism diets. Thus, by applying complementary methods—stable isotope and stomach content composition—we examined the trophic structure of a tropical ecosystem affected by shrimp bottom trawling, aiming to evaluate the importance of the shrimp species as food to coastal fauna and how the fishery exploitation of these resource may affect the ecosystem trophic functioning.

Firstly, some considerations should be made before the interpretation of our results. Although we have used most data from the study area and similar periods, we also utilized stomach content data from the literature, as proxy of the diet of some local species, which did not allow a direct comparison between methods (SCA and SIA), but rather a complementary approach. In addition, we decided not to apply the models to quantify the source importance in isotope approach (e.g., bayesian mixing model), given that our sampling did not take into account some of the known basal sources and benthic invertebrates, which could lead to potential misinterpretation of our results and conclusion as reported by [96]. Therefore, the results presented here are not intended to exhaustively describe the trophic dynamic of the study, but, despite their limitations, we were able to identify the predator and prey groups with major roles in the food-web, and how they could influence the ecosystem trophic dynamic in response to the shrimp fishery in Sirinhaém, northeast Brazil.

Differences on isotopic ratios occurred between SOM and POM. These variations among basal sources are expected [97] and reflects, for example, different contributions to organic deposition in coastal sediments [98–100], which can be seasonally intensified with the increase of fluvial discharges during periods of heavy precipitation [101]. These differences allow the discrimination of two trophic pathways based on benthic or pelagic sources [102]. However, it usually can result in high range of isotopes ratios, given the high diversity of trophic guilds, [103,104]. In general, we found differences and similarities between SCA and the SIA approaches. For example, for shrimps and species of high δ^15^N values, mostly piscivorous and zoobenthivores, the two approached converged. However, we noticed some mismatches in our results for some zooplanktivore (e.g., *O*. *oglinum*), omnivore (e.g., *C*. *ornatus* and *C*. *danae*) and zoobenthivores species (e.g., *B*. *marinus*, *L*. *synagris* and *Bairdiella ronchus*). Generalist trophic habits associated with omnivores that feed on multiple trophic levels and taxonomic groups, introduce considerable uncertainty into diet patterns by SCA and SIA [105], mainly related to age-dependent trophic shifts [106]. Some studies report wide variations and even lack of correlation between SIA and SCA approaches [35,39,42], mainly related to aspects of differential size range [107], life stage [105], season [108], isotopic fractionation [109] and spatial-temporal scale [34].

For some zoobenthivores, isotopic niches often overlap with piscivorous [110], reflecting the opportunistic behavior of this group in an environment where food sources are highly available. Zoobenthivore fishes had wide feeding preferences [65,111], which would possibly provide large variations of δ^15^N composition [112,113]. However, the nitrogen ratios for this group slightly varied, indicating that they feed on food sources that have similar isotopic composition, consisting mostly of penaeid shrimps, small crabs and fishes in lower proportion. The availability and consequently the aggregation of prey can strongly influence the species feeding habitat patterns [114,115]; the predator would feed on prey largely available. Penaeidae shrimps are widely explored in the region, particularly the seabob shrimp (*X*. *kroyeri*), the most abundant one, and the pink (*P*. *subtilis*) and white shrimp (*P*. *schmitti*), with high market-values [62]. Although we have not evaluated the worms isotopic compositions, fish diet revealed a relative high contribution of this taxonomic group, mostly polychaets for some species (e.g., *Eucinostomus argenteus*–present study and *Symphurus tesselatus—*Guedes et al. [84]). Thus, polychaets should be considered as an additional important source of energy for the higher trophic levels.

Our findings with two complementary tools (SCA and SIA) helped to understand the contribution of benthic sources, the importance of crustaceans, especially shrimps, in transporting energy from food web base to upper trophic levels and bycatch species of high δ^15^N values, such as the top predators (e.g., *I*. *parvipinis* and *C*. *nobilis*), thus providing support to coastal food-web in Sirinhaém. The importance of the benthic community for the trophic functioning of the coastal zone, specifically crustaceans, has been reported in other ecosystems affected by bottom trawl fishing, for example, in southeast Brazil [116–120], and in other parts of the world, such as Australia [121], Irish Sea [24] and North Sea [122]. The presence of large mud banks in these coastal areas, which usually favors large occurrences of benthic invertebrates, such as worms and crustaceans, explains this huge importance. In our study case, the fishing area in Sirinhaém is close to river mouth with depths ranging from 4 to 20 m, the seabed is composed of sand and predominantly mud zones, where most of the organisms and fishing effort is homogeneously concentrated. Hinz et al. [45] highlighted the negative effect of fishery trawling, removing not only fish and benthos, but also changing prey and predator relationships. The resuspension of sediment from trawling may cause death of a wide range of benthic organism [13], including benthic invertebrate preys of major role in energy transfer for the food-web, as for example in our case, the shrimps (e.g., *X*. *kroyeri*, *P*. *subtilis* and *P*. *schmitti*), crabs (e.g., *C*. *ornatus* and *C*. *danae*) and worms. The food-web dependence of the benthic invertebrates should also be considered in ecosystem approach to fisheries, since any regulation may therefore have consequences on both benthic prey and the consumers [45,123].

Specifically in Sirinhaém, since there are no fishing regulations [59], the cumulative effects of trawling on population parameters (e.g., size and food intake), species composition [124,125], potential decreasing the abundance of benthic preys and fish species may lead to intense changes in the trophic structure of the ecosystem, which may cause the trophic cascade effect (top-down or bottom-up) and potentially affect the food web and the sustainability of the fishery.

## Supporting information

S1 Table Complementary sampling information. Mean, minima, maxima size, number of samples (n) in each quarter/year by species/group considered off the Sirinhaém coast, northeastern Brazil. For fish the size is related to standard length (cm); *for shrimps, carapace length (cm) and ** for mollusk, mantle length (cm).
(DOCX)

S2 Table Additional diet data information considered to present study off the Sirinhaém coast, northeastern Brazil. Location and year of data, total length range used and whether seasonal or ontogenic characteristics were considered (yes (y) or no (n)).
(DOCX)
