# Supplementary material for: Trophic structure of a nektobenthic community exploited by a multispecific bottom trawling fishery in Northeastern Brazil
Source: PLoS One. 2021 Feb 8;16(2):e0246491. doi: 10.1371/journal.pone.0246491 (PMC7870051; doi:10.1371/journal.pone.0246491)
Supplement: S1 Table — Mean, minima, maxima size, number of samples (n) in each quarter/year by species/group considered off the Sirinhaém coast, northeastern Brazil. For fish the size is related to standard length (cm); *for shrimps, carapace length (cm) and ** for mollusk, mantle length (cm). (DOCX) [file pone.0246491.s001.docx]

S1 Table

|  |  |  |  | **2013** | | **2014** | | | **2015** | | | | **2019** |
| --- | --- | --- | --- | --- | --- | --- | --- | --- | --- | --- | --- | --- | --- |
| **Groups/species** | **Code** | **n** | **Mean size [min – max] (cm)** | **third quarter** | **fourth quarter** | **first quarter** | **second quarter** | **third quarter** | **first quarter** | **second quarter** | **third quarter** | **fourth quarter** | **fourth quarter** |
| **Basal sources** |  |  |  |  |  |  |  |  |  |  |  |  |  |
| Sedimentary organic matter | SOM | 8 | - |  |  |  |  |  | 6 |  | 2 |  |  |
| *Lobophora variegata* | lob.var | 6 | - |  |  |  |  |  | 3 |  | 3 |  |  |
| *Gracilaria cervicornis* | gra.cer | 6 | - |  |  |  |  |  | 3 |  | 3 |  |  |
| *Sargassum* sp. | sar.sp | 6 | - |  |  |  |  |  | 3 |  | 3 |  |  |
| Particulate organic matter | POM | 5 | - |  |  |  |  |  | 3 |  | 2 |  |  |
| **Invertebrates** |  |  |  |  |  |  |  |  |  |  |  |  |  |
| Zooplankton | zoo | 6 | - |  |  |  |  |  | 3 |  | 3 |  |  |
| **Penaeus subtilis* | pen.sub | 14 | 2.55 [1.9 to 3.3] |  | 3 | 5 |  |  | 3 |  | 3 |  |  |
| **Penaeus schmitti* | pen.sch | 20 | 3.31 [2 to 4.1] |  | 4 | 6 | 4 |  | 2 |  | 4 |  |  |
| **Callinectes danae* | cal.dan | 5 | 6.1 [5.9 to 6.3] |  |  |  |  |  |  |  | 5 |  |  |
| **Callinectes ornatus* | cal.orn | 3 | 4.9 [4.8 to 5.1] |  |  |  |  |  | 3 |  |  |  |  |
| **Xiphopenaeus kroyeri* | xip.kro | 17 | 1.76 [1 to 2.1] | 2 |  | 7 |  |  | 5 |  | 3 |  |  |
| ***Lolliguncula brevis* | log.bre | 5 |  |  |  |  |  |  |  |  |  |  | 5 |
| **Fishes** |  |  |  |  |  |  |  |  |  |  |  |  |  |
| *Citharichthys spilopterus* | Cit.spi | 3 | 11.1 [8.9 to 13.2] |  |  |  |  |  | 1 |  | 2 |  |  |
| *Diapterus auratus* | Dia.aur | 7 | 12.94 [10.5 to 17.5] |  | 1 |  |  |  |  |  | 5 | 1 |  |
| *Opisthonema oglinum* | Opi.ogl | 8 | 15.08 [9.4 to 17] |  |  | 6 |  | 2 |  |  |  |  |  |
| *Symphurus tessellatus* | Sym.tes | 6 | 15.01 [14.1 to 16.2] |  |  |  |  |  | 3 |  | 3 |  |  |
| *Diapterus rhombeus* | Dia.rho | 8 | 10.27 [10.2 to 10.4] |  |  |  |  | 5 | 2 |  |  | 1 |  |
| *Lutjanus synagris* | Lut.syn | 6 | 13.03 [7.7 to 19] |  |  |  |  |  |  | 3 | 3 |  |  |
| *Bairdiella ronchus* | Bai.ron | 3 | 11.23 [11 to 11.4] |  |  |  |  |  |  | 3 |  |  |  |
| *Chirocentrodon bleekerianus* | Chi.ble | 4 | 10.06 [10.3 to 10.9] |  |  |  |  |  |  |  |  |  | 4 |
| *Eucinostomus argenteus* | Euc.arg | 14 | 8.52 [6.5 to 11.9] |  |  | 3 |  |  |  | 11 |  |  |  |
| *Bagre bagre* | Bag.bag | 3 | 9.67 [7.9 to 13] |  |  |  |  |  |  |  |  | 3 |  |
| *Caranx hippos* | Car.hip | 8 | 16.8 [16.5 to 17.2] |  |  |  |  |  |  |  |  | 8 |  |
| *Micropogonias furnieri* | Mic.fur | 7 | 25.45 [24.5 to 26.8] |  | 1 | 4 | 1 | 1 |  |  |  |  |  |
| *Bagre marinus* | Bag.mar | 8 | 9.13 [7.1 to 12] |  |  | 5 |  |  | 3 |  |  |  |  |
| *Larimus breviceps* | Lar.bre | 3 | 12.00 [9.6 to 13.7] |  |  |  |  |  |  |  |  |  | 3 |
| *Stellifer microps* | Ste.mic | 4 | 12.02 [11.3 to 13.5] |  |  |  |  |  |  |  |  |  | 4 |
| *Isopisthus parvipinnis* | Iso.par | 4 | 10.25 [9.1 to 13.6] |  |  |  |  |  |  |  |  |  | 4 |
| *Conodon nobilis* | Con.nob | 4 | 9.57 [7.4 to 10.9] |  |  |  |  |  |  |  |  |  | 4 |
| *Paralonchurus brasiliensis* | Par.bra | 3 | 14.33 [11 to 20.1] |  |  |  |  |  |  |  |  |  | 3 |
