# Supplementary material for: Trophic structure of a nektobenthic community exploited by a multispecific bottom trawling fishery in Northeastern Brazil
Source: PLoS One. 2021 Feb 8;16(2):e0246491. doi: 10.1371/journal.pone.0246491 (PMC7870051; doi:10.1371/journal.pone.0246491)
Supplement: S2 Table — Location and year of data, total length range used and whether seasonal or ontogenic characteristics were considered (yes (y) or no (n)). (DOCX) [file pone.0246491.s002.docx]

S2 Table

| Species | Cod | Site | n | Total length (cm) | Year | Seasonality (y/n) | Ontogeny (y/n) | Source |
| --- | --- | --- | --- | --- | --- | --- | --- | --- |
| *Bagre bagre* | bag.bag | Maranhão, Brazil | - | - | - | - | - | (Pinheiro-Sousa et al. 2015) |
| *Bagre marinus* | bag.mar | Pernambuco, Brazil | 105 | [17.40 ± 9.9 cm] | 2013-2014 | n | n | our data |
| *Bairdiella ronchus* | bai.ron | Pernambuco, Brazil | 62 | [16.68 ± 1.8 cm] | 2013 | n | n | our data |
| *Caranx hippos* | car.hip | Pernambuco, Brazil | 15 | [14.18 ± 1.7 cm] | 2013 | n | n | our data |
| *Chirocentrodon bleekerianus* | chi.ble | Sao Paulo, Brazil | - | - | - | - | - | (Muto et al. 2008) |
| *Citharichthys spilopterus* | cit.spi | Rio de Janeiro, Brazil | - | - | - | - | - | (Guedes et al. 2004) |
| *Conodon nobilis* | con.nob | Pernambuco, Brazil | 165 | [13.36 ± 3.3 cm] | 2011-2012 | y | y | our data |
| *Diapterus auratus* | dia.aur | Pernambuco, Brazil | 74 | [17.22 ± 5.6 cm] | 2013-2014 | n | n | our data |
| *Diapterus rhombeus* | dia.rho | Pernambuco, Brazil | 25 | [8.50 ± 2.0 cm] | 2013-2014 | n | n | our data |
| *Eucinostomus argenteus* | euc.arg | Pernambuco, Brazil | 332 | [8.62 ± 3,8 cm] | 2013-2014 | y | y | our data |
| *Isopisthus parvipinnis* | iso.par | Pernambuco, Brazil | 69 | [14.50 ± 3.6 cm] | 2011-2012 | n | n | our data |
| *Larimus breviceps* | lar.bre | Rio de Janeiro, Brazil | - | - | - | - | - | (Bessa et al. 2014) |
| *Lutjanus synagris* | lut.syn | Rio Grande do Norte, Brazil | - | - | - | - | - | (Costa 2013) |
| *Micropogonias furnieri* | mic.fur | Rio de Janeiro, Brazil | - | - | - | - | - | (Freret & Vanderli 2003) |
| *Opisthonema oglinum* | opi.ogl | Sao Paulo, Brazil | - | - | - | - | - | (Caludio Höfling et al. 1998) |
| *Paralonchurus brasiliensis* | par.bra | Pernambuco, Brazil | 72 | [13.60 ± 2,3 cm] | 2011-2012 | n | n | our data |
| *Stellifer microps* | ste.mic | Pernambuco, Brazil | 145 | [11.46 ± 2,2 cm] | 2011-2014 | y | y | our data |
| *Symphurus tessellatus* | sym.tes | Rio de Janeiro, Brazil | - | - | - | - | - | (Guedes et al. 2004) |
| *Callinectes danae* | cal.dan | Santa Catarina, Brazil | - | - | - | - | - | (Branco & Verani 1997) |
| *Callinectes ornatus* | cal.orn | Santa Catarina, Brazil | - | - | - | - | - | (Olinto Branco et al. 2002) |
| *Lolliguncula brevis* | lol.bre | São Paulo, Brazil | - | - | - | - | - | (Coelho et al. 2010, ZALESKI 2010) |
| *Penaeus schmitti* | pen.sch | Pernambuco, Brazil | 36 | [8.91 ± 2,0 cm] | 2018-2019 | y | n | our data |
| *Penaeus subtilis* | pen.sub | Pernambuco, Brazil | 45 | [9.50 ± 2,2 cm] | 2018-2019 | y | n | our data |
| *Xiphopenaeus kroyeri* | xip.kro | Pernambuco, Brazil | 117 | [6.98 ± 1.3 cm] | 2018-2019 | y | n | our data |
| Zooplankton | zoo | - | - | - | - | - | - |  |
